# Supplementary material for: How breakthroughs happen: Unearthing the boundary conditions of eco-friendly deliberate practice and eco-innovation performance
Source: PLoS One. 2025 Jan 10;20(1):e0316802. doi: 10.1371/journal.pone.0316802 (PMC11723645; doi:10.1371/journal.pone.0316802)
Supplement: S1 Appendix — (DOCX) [file pone.0316802.s001.docx]

**Appendix I:** Measurement Scales

**Perceived Organizational Support (POS)**
(Adapted from Eisenberger et al., 1986)

1. My organization strongly considers my goals and values.
2. My organization cares about my well-being.
3. My organization is willing to help me when I need a special favor.
4. My organization cares about my opinions.
5. My organization takes pride in my accomplishments.
6. My organization shows very little concern for me. (Reverse-coded)
7. My organization would forgive an honest mistake on my part.
8. My organization values my contribution to its well-being.

**Developmental Leadership (DL)**
(Adapted from House, 1998)

1. Encourages staff to improve their job-related skills.
2. Provides feedback to help staff develop professionally.
3. Supports career advancement and development.

**Eco-Friendly Deliberate Practice (EDP)**
(Adapted from Sonnentag & Irion, 2010)

1. In order to improve my eco-innovation skills, I deliberately take some time to re-think my working technique.
2. I seek feedback on how to improve eco-innovation skills.
3. I regularly reflect on my eco-friendly work practices.
4. I engage in eco-innovation tasks even when it is difficult.
5. I set goals to enhance my eco-innovation performance.
6. I actively look for ways to increase my eco-friendly effectiveness.
7. I continuously work on improving my eco-innovation abilities.
8. I make a deliberate effort to apply new eco-innovation knowledge.
9. I plan specific ways to improve my eco-innovation output.
10. I experiment with different eco-innovation techniques to improve performance.
11. I consciously adjust my working style to enhance eco-friendliness.
12. I focus on tasks that challenge my eco-innovation skills.
13. I spend extra time improving my eco-innovation processes.
14. I frequently review my progress in eco-innovation tasks.
15. I engage in eco-friendly practices to help my organization achieve its environmental goals.

**Eco-Innovation Performance (EP)**
(Adapted from Welbourne et al., 1998)

1. Creates better eco-friendly processes and routines.
2. Generates new eco-innovation ideas that are beneficial to the organization.
3. Improves the sustainability of the organization's operations.
4. Actively contributes to the organization’s environmental initiatives.

**Employee Resilience (ER)**
(Adapted from Al-Omar et al., 2019)

1. I tend to bounce back quickly after hard times.
2. I can adapt easily when confronted with environmental challenges.
3. I stay focused and positive when faced with eco-innovation difficulties.
4. I recover well from setbacks related to eco-innovation performance.
5. I feel confident in my ability to handle unforeseen eco-friendly challenges.
6. I persist through eco-friendly projects even when faced with major obstacles.
