# Supplementary material for: How breakthroughs happen: Unearthing the boundary conditions of eco-friendly deliberate practice and eco-innovation performance
Source: PLoS One. 2025 Jan 10;20(1):e0316802. doi: 10.1371/journal.pone.0316802 (PMC11723645; doi:10.1371/journal.pone.0316802)
Supplement: Attachment — Submitted filename: Response letter.docx [file pone.0316802.s003.docx]

PONE-D-23-14796

How breakthroughs happen: Unearthing the boundary conditions of eco-friendly deliberate practice and eco-innovation performance

PLOS ONE

Dear Dr.,

Thank you for submitting your manuscript to PLOS ONE. After careful consideration, we feel that it has merit but does not fully meet PLOS ONE’s publication criteria as it currently stands. Therefore, we invite you to submit a revised version of the manuscript that addresses the points raised during the review process.

The author needs to provide approval from the ethics committee.

The final decision is to integrate the suggestions of the two reviewers.

We look forward to receiving your revised manuscript.

Kind regards,

Liping Liu
Academic Editor
PLOS ONE

Dear Dr. Liu,

Thank you for your valuable feedback and for providing the opportunity to revise our manuscript, *"How breakthroughs happen: Unearthing the boundary conditions of eco-friendly deliberate practice and eco-innovation performance"*. We appreciate the reviewers' insightful comments, which have helped improve the clarity and quality of the manuscript.

We have addressed all the points raised by the reviewers, including simplifying the abstract, refining the introduction, providing clearer explanations of key concepts, and incorporating a more detailed discussion of the theoretical implications. Additionally, we have moved the tables from the appendix to the main body for clarity, as suggested. We will also ensure to provide the approval from the ethics committee as requested.

We look forward to submitting the revised version and are confident that the revisions have strengthened the manuscript.

Thank you for your time and consideration.

Kind regards,

Journal requirements:

When submitting your revision, we need you to address these additional requirements.

1. Please ensure that your manuscript meets PLOS ONE's style requirements, including those for file naming. The PLOS ONE style templates can be found at
<https://journals.plos.org/plosone/s/file?id=wjVg/PLOSOne_formatting_sample_main_body.pdf> and
<https://journals.plos.org/plosone/s/file?id=ba62/PLOSOne_formatting_sample_title_authors_affiliations.pdf>

2. Note from Emily Chenette, Editor in Chief of PLOS ONE, and Iain Hrynaszkiewicz, Director of Open Research Solutions at PLOS: Did you know that depositing data in a repository is associated with up to a 25% citation advantage (<https://doi.org/10.1371/journal.pone.0230416>)? If you’ve not already done so, consider depositing your raw data in a repository to ensure your work is read, appreciated and cited by the largest possible audience. You’ll also earn an Accessible Data icon on your published paper if you deposit your data in any participating repository (<https://plos.org/open-science/open-data/#accessible-data>).

3. Thank you for stating the following financial disclosure: 
 [This article is one of the interim results of the Jilin Provincial Education Department's 2022 Fund Project "Research on the Construction System of Jilin Province's Long-Term Care Talent Team (JJKH20230862SK)" and the 2022 Changchun Normal University Humanities and Social Sciences Fund Project "Comparative Study on the Construction System of Long-Term Care Talent Teams in China and Japan (CSJJ2022010SK)".].  
Please state what role the funders took in the study.  If the funders had no role, please state: "The funders had no role in study design, data collection and analysis, decision to publish, or preparation of the manuscript." 
If this statement is not correct you must amend it as needed. 
Please include this amended Role of Funder statement in your cover letter; we will change the online submission form on your behalf.

4. In the online submission form, you indicated that [Data is available upon reasonable request from the corresponding author.]. 
All PLOS journals now require all data underlying the findings described in their manuscript to be freely available to other researchers, either 1. In a public repository, 2. Within the manuscript itself, or 3. Uploaded as supplementary information.
This policy applies to all data except where public deposition would breach compliance with the protocol approved by your research ethics board. If your data cannot be made publicly available for ethical or legal reasons (e.g., public availability would compromise patient privacy), please explain your reasons on resubmission and your exemption request will be escalated for approval.

5. Please amend your authorship list in your manuscript file to include author Yin-shi Jin, Shahid Iqbal, Tehreem Fatima, and Arslan Ayub.

**Reviewers' comments:

Reviewer's Responses to Questions**

**Comments to the Author**

1. Is the manuscript technically sound, and do the data support the conclusions?

The manuscript must describe a technically sound piece of scientific research with data that supports the conclusions. Experiments must have been conducted rigorously, with appropriate controls, replication, and sample sizes. The conclusions must be drawn appropriately based on the data presented.

Reviewer #1: Partly

Reviewer #2: Yes

2. Has the statistical analysis been performed appropriately and rigorously?

Reviewer #1: Yes

Reviewer #2: Yes

3. Have the authors made all data underlying the findings in their manuscript fully available?

The [PLOS Data policy](http://www.plosone.org/static/policies.action#sharing) requires authors to make all data underlying the findings described in their manuscript fully available without restriction, with rare exception (please refer to the Data Availability Statement in the manuscript PDF file). The data should be provided as part of the manuscript or its supporting information, or deposited to a public repository. For example, in addition to summary statistics, the data points behind means, medians and variance measures should be available. If there are restrictions on publicly sharing data—e.g. participant privacy or use of data from a third party—those must be specified.

Reviewer #1: No

Reviewer #2: Yes

4. Is the manuscript presented in an intelligible fashion and written in standard English?

PLOS ONE does not copyedit accepted manuscripts, so the language in submitted articles must be clear, correct, and unambiguous. Any typographical or grammatical errors should be corrected at revision, so please note any specific errors here.

Reviewer #1: Yes

Reviewer #2: Yes

5. Review Comments to the Author

**Reviewer #1:** Comment 1: The introduction is lengthy and contains numerous complex sentences. Consider simplifying the language for better readability. Ensure that each sentence contributes significantly to the overall understanding of the study.

Comment 2: The identification of the literature gap is well-established, emphasizing the scarcity of empirical studies on the boundary conditions of eco-innovation performance. However, it might be helpful to briefly mention the specific gaps this study aims to address within the existing literature.

Comment 3: The final paragraph seems in introduction incomplete and lacks a concluding sentence. Consider summarizing the key points discussed in the introduction and providing a segue into the subsequent sections of the paper.

Comment 4: The manuscript introduces several concepts such as POS, DL, EDP, EP, and ER. While these are explained individually, a summary or conceptual framework that visually illustrates the relationships among these constructs could enhance clarity for the reader.

Comment 5: The manuscript draws on various theories (e.g., social cognitive theory, path-goal theory, transformational leadership theory) to support the proposed relationships. It would be beneficial to provide a more integrated discussion of how these theories complement each other in explaining the proposed relationships.

Comment 6: The authors state that data were collected through face-to-face interviews, but details on the interviewers' training, standardization, and monitoring are missing. Providing such information would enhance the study's transparency and reliability.

Comment 7: While the authors provide sample items for each construct, it would be beneficial to include the full set of items in an appendix or supplementary material. This would aid readers in assessing the comprehensiveness and appropriateness of the measurement instruments.

Comment 8: The scale for EDP, adapted from Sonnentag and Irion (2010), contains 15 items. Considering the potential respondent burden, the authors may want to discuss the rationale behind using such a lengthy scale and how they ensured respondents' engagement and accuracy.

Comment 9: The discussion outlines the main contribution as exploring the antecedents and outcomes of EDP in the service industry in Pakistan. However, the authors could provide more insight into the broader implications of these findings. How does this study contribute to the existing knowledge in the field of eco-innovation and employee performance? Are there practical implications for organizations or policymakers?

Comment 10: In the theoretical implications the use of social cognitive theory to explain the mediating role of EDP is mentioned, but the discussion lacks a nuanced explanation of how this theory precisely operates within the proposed model. Clarifying the underlying mechanisms and processes through which mastery experiences and self-efficacy are accumulated would enhance the theoretical coherence of the argument.

Comment 11: The practical implications lack specificity and concrete guidance for organizations. While the study suggests interventions such as role clarity, job embeddedness, and flexible working arrangements to promote EDP, it would be more beneficial to provide detailed and actionable strategies that organizations can implement. Clear examples and case studies could enhance the practical utility of the recommendations.

**Response**

**Comment 1**: We have revised the introduction, simplifying complex sentences and ensuring that each sentence contributes to the overall understanding of the study.

**Comment 2**: The introduction now briefly mentions the specific gaps this study aims to address, focusing on the boundary conditions of eco-innovation performance.

**Comment 3**: The final paragraph of the introduction has been revised to include a summary of key points and a clear segue into the next sections.

**Comment 4**: A conceptual framework summarizing the relationships among POS, DL, EDP, EP, and ER has been included for clarity.

**Comment 5**: We have integrated the discussion of social cognitive theory, path-goal theory, and transformational leadership theory to explain how these frameworks complement each other in supporting the proposed relationships.

**Comment 6**: We clarified that the study is questionnaire-based, not interview-based, and have included details on the standardization of the questionnaire process.

**Comment 7**: The full set of measurement items has been provided in Appendix I to ensure comprehensiveness and transparency of the instruments.

**Comment 8**: The rationale for using the 15-item EDP scale, along with measures to maintain respondent engagement, has been discussed in the methodology section.**Comment 9**: We have expanded the discussion to explain the broader implications of the study for the fields of eco-innovation and employee performance, and included practical implications for organizations and policymakers.

**Comment 10**: The theoretical implications have been clarified, with a more detailed explanation of how social cognitive theory operates within the model, focusing on mastery experiences and self-efficacy.

**Comment 11**: The practical implications have been revised to include specific, actionable strategies for organizations, along with examples to enhance their practical utility.

**Reviewer #2:** The paper is well written. However, I would like to provide some suggestions to improve the quality of this paper.

The abstract is well written, well composed. However, breakdown complex words and sentences like predicated on deliberate practice" and "nexus."

The introduction is quite lengthy. Consider combining related ideas into shorter paragraphs.
Define key terms like eco-innovation performance (EP) and employee resilience (ER) earlier in the introduction.
Highlight the specific research gap and the study's contribution more prominently.

The methodology is well composed. The data analysis is complete. However, tabulation must be in place, these are in the appendix section. Place them where they belong to make things more clear.
In the conclusion; summarise the key findings in a shorter and more impactful way. You can remove sentences that repeat information already stated like "underexplored construct" and "hitherto unexplored mechanism."
Briefly define EDP and EP for readers who might not be familiar with these acronyms (e.g., employee-driven innovation for EDP, environmental performance for EP).

**Response**

**Abstract**: We have simplified complex words and phrases like "predicated on deliberate practice" and "nexus" in the abstract for better readability.

**Introduction Length**: The introduction has been shortened by combining related ideas into more concise paragraphs for clarity.

**Key Terms Definition**: Key terms like eco-innovation performance (EP) and employee resilience (ER) have been defined earlier in the introduction.

**Research Gap and Contribution**: The research gap and the study's contributions have been highlighted more prominently in the introduction.

**Tabulation**: The tables have been moved from the appendix to the main body of the paper to improve clarity and coherence.
