# Supplementary material for: How breakthroughs happen: Unearthing the boundary conditions of eco-friendly deliberate practice and eco-innovation performance
Source: PLoS One. 2025 Jan 10;20(1):e0316802. doi: 10.1371/journal.pone.0316802 (PMC11723645; doi:10.1371/journal.pone.0316802)
Supplement: Attachment — Submitted filename: Response letter 2.docx [file pone.0316802.s004.docx]

**PONE-D-23-14796R1
How breakthroughs happen: Unearthing the boundary conditions of eco-friendly deliberate practice and eco-innovation performance
PLOS ONE

Dear Dr. Ayub,

Thank you for submitting your manuscript to PLOS ONE. After careful review, we find that your work holds potential; however, it does not fully meet PLOS ONE’s publication criteria in its current form. We would like to offer you the opportunity to submit a revised version that addresses the following points raised during the editorial and peer review process:

•       To ensure the manuscript adheres to ethical guidelines, we require you to provide an official document of Institutional Review Board (IRB) approval for the study. This document is necessary for us to confirm that the research was conducted ethically and in compliance with relevant standards.
•       It has been observed that the manuscript lacks recent literature to support your discussion and analysis. To enhance the robustness of your work, we request that you include studies published in 2024 that align with and reinforce your research context and findings.

Please submit the revised manuscript along with the requested documentation by 17-12-2024 (within one week). Ensure that the response to the changes and a point-by-point explanation of the revisions are included with your resubmission.

Should you have any questions regarding the revision process or the comments provided, please feel free to reach out to us.
We look forward to receiving your revised manuscript.


Regards,

Ali Junaid Khan, PhD
Academic Editor
PLOS ONE**

***Response***

*Thank you for acknowledging our study and recommending the inclusion of the latest references from 2024. We have enriched the manuscript with relevant and recent citations, as per your suggestion, to strengthen its foundation. Additionally, we have attached the ethical approval document for your reference. We trust these updates align with your expectations and enhance the overall quality of the study.*
